# Sleep and Drowsiness in Cattle: A Narrative Review of Physiology, Measurement, and Methodological Challenges

**DOI:** 10.3390/ani16203279

**Published:** 2026-10-17

**Authors:** Pedro H. F. Torres, Fernanda F. Alves, Maria Eduarda M. Lopes, Gercino F. Virgínio Júnior, Bruce F. O’Hara, Kevin D. Donohue, Daniel H. M. Watanabe, Danilo D. Millen

**Affiliations:** 1 School of Agricultural and Veterinary Sciences, São Paulo State University (UNESP), Jaboticabal 14884-900, SP, Brazilmem.lopes@unesp.br (M.E.M.L.);; 2 Department of Biology, University of Kentucky, Lexington, KY 40508-0225, USA; bohara@uky.edu; 3 Department of Electrical and Computer Engineering, University of Kentucky, Lexington, KY 40508-0225, USA; kevin.donohue1@uky.edu

**Keywords:** accelerometry, cattle, drowsing, polysomnography, rumination, sleep

## Abstract

Sleep in cattle is a complex physiological state comprising non-rapid eye movement (NREM) and rapid eye movement (REM) sleep, while drowsing represents an intermediate vigilance state between wakefulness and sleep. Research has progressed from invasive electrophysiological recordings to noninvasive polysomnography (PSG), behavioral assessment, accelerometry, and automated classification approaches. However, accurately identifying and quantifying vigilance states under production conditions remains challenging. This narrative review, based on 22 primary studies identified through searches of Web of Science, Scopus and Google Scholar, critically synthesizes current knowledge of sleep physiology in cattle, the approaches used to assess it, and the principal methodological limitations affecting interpretation of the available evidence. Cattle exhibit a polyphasic sleep pattern, with NREM generally accounting for the largest proportion of total sleep. However, reported sleep and drowsing durations vary considerably among studies, and these differences may reflect both biological variation and methodological factors. Drowsing is particularly difficult to characterize because its behavioral and physiological features overlap with quiet wakefulness and light sleep. Rumination represents an additional challenge because associated muscular activity can interfere with electrophysiological recordings and complicate vigilance-state classification. Behavioral observations and accelerometry provide scalable measures of resting and sleep-like behavior but cannot independently establish physiological sleep, whereas PSG provides greater physiological resolution while remaining susceptible to artifacts and practical constraints. Current evidence is insufficient to establish causal relationships between sleep variation and cattle health, welfare, or productive performance. Future research should improve physiological classification during overlapping behavioral states, better validate scalable monitoring approaches, and evaluate the functional consequences of sleep variation across diverse cattle populations and production conditions.

## Simple Summary

Sleep is a fundamental biological state in mammals, but knowledge of sleep in cattle remains limited. This review summarizes current evidence on sleep and drowsing in cattle and critically examines how researchers identify and measure these states. The available evidence comes from a small number of studies, conducted mainly with dairy cows under controlled or confined conditions. Cattle exhibit distinct vigilance states, including wakefulness, drowsing, non-rapid eye movement sleep, and rapid eye movement sleep. However, accurately distinguishing these states can be difficult, particularly because resting behavior does not necessarily indicate physiological sleep and rumination can interfere with physiological recordings. Behavioral observations and wearable sensors offer practical approaches for monitoring animals, whereas physiological recordings better distinguish vigilance states but also face important methodological limitations. Consequently, reported differences in sleep duration among studies may reflect both biological variation and differences in measurement and classification methods. Current evidence is insufficient to determine how sleep variation affects cattle health, welfare, or productive performance. Further research using physiologically validated approaches across diverse cattle populations, management systems, and environmental conditions is needed to establish the biological and practical relevance of sleep in cattle.

## 1. Introduction

Sleep is a naturally occurring physiological state characterized by reduced wakefulness, motor activity, and metabolic rate [1,2]. In humans, it is widely recognized as an indicator of health, welfare, and functional capacity [3]. Although its mechanisms are well described in humans and laboratory rodents, conserved features of sleep regulation across mammals suggest that it may also be a candidate biological marker in cattle, potentially reflecting adaptation to management and production conditions [4].

Research on sleep in cattle has progressed since the first electrophysiological descriptions [5,6], yet important gaps remain. Available evidence comes from a relatively small number of studies and spans a limited range of animal categories, management systems, and environmental conditions. In addition, drowsing remains less clearly defined than NREM and REM sleep and represents an important source of uncertainty in vigilance-state classification. Rumination further complicates sleep assessment because it can overlap with periods of reduced vigilance and interfere with physiological recordings. These limitations make it difficult to determine how much of the variation in reported sleep patterns reflects biological differences rather than differences in measurement and classification.

Beyond describing sleep architecture, an important unresolved question is whether sleep variation has functional consequences for cattle. In mammals, sleep is associated with endocrine pathways involved in appetite and energy balance [7] and immune modulation [8,9]. Cattle under production conditions also face nutritional, environmental, and social challenges that can alter physiological state [10,11]. These observations provide biological plausibility for relationships between sleep and physiological function; however, direct causal relationships between sleep variation and health, welfare, or productive performance remain unestablished in cattle. Distinguishing such biological hypotheses from demonstrated effects is therefore essential when interpreting the current evidence.

This review aims to: (i) synthesize current knowledge of sleep physiology in cattle, including non-rapid eye movement (NREM) sleep, rapid eye movement (REM) sleep, and drowsing; (ii) critically evaluate approaches used to assess sleep, from behavioral observation to polysomnography and wearable technologies; (iii) examine methodological limitations and sources of uncertainty, particularly behavioral overlap, rumination, and physiological signal artifacts; and (iv) identify the major biological and methodological questions that must be addressed to determine the functional relevance of sleep in cattle.

## 2. Methodology

This narrative review synthesizes existing knowledge on sleep in cattle, its vigilance states, and the methodologies used to assess them. To this end, we searched Web of Science, Scopus, and Google Scholar to identify studies investigating sleep and related vigilance states in cattle. We used different keyword combinations to increase both the sensitivity and specificity of the searches.

The search strategies were divided into two categories: basic and advanced. The basic strategy aimed to provide a broad overview of the literature, while the advanced strategy refined the search and identified more specific studies. The basic combination was: (“cattle sleep”) OR (“bovine sleep”) OR (“cow sleep”). The advanced combination was: (“sleep” OR “drowsing” OR “drowsiness” OR “REM sleep” OR “NREM sleep” OR “non-rapid eye movement” OR “slow wave sleep” OR “circadian rhythm”) AND (“cattle” OR “bovine” OR “dairy cow” OR “dairy cattle” OR “beef cattle” OR “calf” OR “calves”) AND (“polysomnography” OR “EEG” OR “electroencephalography” OR “electrooculography” OR “EOG” OR “electromyography” OR “EMG” OR “accelerometer” OR “actigraphy” OR “machine learning”). These search strings were applied in Scopus and Web of Science, whereas Google Scholar was used as a complementary search source to identify additional relevant and foundational studies. In total, the searches yielded 1414 records.

The literature search was initially conducted in 2025 and was updated during manuscript preparation and revision to incorporate newly published studies meeting the eligibility criteria. Duplicate records were removed before screening, and two reviewers evaluated the retrieved records. Potentially relevant studies were subsequently assessed by full-text evaluation against the eligibility criteria. Earlier foundational studies on cattle sleep physiology were also retained when relevant.

Eligibility criteria included articles published in English, experimental studies conducted with dairy or beef cattle (regardless of age), and assessments of sleep or sleep-related vigilance states using physiological recordings, behavioral observations, accelerometry, or other sensor-based approaches. Sleep was considered to be assessed directly when vigilance states were classified from electrophysiological recordings (EEG or electrocorticography combined with EMG or EOG) or when postures or behaviors explicitly described as indicators of sleep, such as sleep-like or sleep-related postures, were quantified by direct observation, video, or accelerometers. Studies based exclusively on posture or accelerometry were eligible even without physiological validation, but their results are interpreted as sleep-related behavior rather than physiological sleep (Section 7.1). Studies were excluded from the primary evidence base when they were not conducted in cattle, did not directly address sleep or sleep-related vigilance states, or assessed only lying, resting, or inactivity without explicitly relating these measures to sleep. Following full-text assessment, 22 articles met the eligibility criteria and constituted the primary evidence base of this review.

No formal quality score was assigned because the designs, measurements, and outcomes of these studies were too heterogeneous for a common appraisal tool. Instead, the sample size, recording duration and repetition, measurement and validation method, treatment of rumination and other unscorable epochs, and experimental setting of each study were extracted (Supplementary Tables S1–S3) and considered when interpreting the evidence: physiological recordings were given precedence over posture-based measures, and effects that were replicated or obtained by experimental manipulation over findings from single observational studies (Section 8).

The additional literature that did not constitute primary evidence on sleep in cattle was used where necessary to provide physiological context, explain mechanisms, or support the interpretation of methodological issues and broader concepts not directly addressed by the primary studies. These references were considered the supporting literature and were not included among the 22 primary studies.

## 3. Why Measure Sleep in Cattle?

Sleep is closely associated with health and physiological function in humans [3]. Although its physiological functions are well characterized in humans and laboratory models such as rodents, much of this knowledge remains concentrated in these species. However, conserved mechanisms of sleep regulation and associated physiological responses across mammals [4] provide a biological basis for investigating whether variation in sleep reflects physiological responses to environmental and management conditions in cattle.

Sleep in cattle has increasingly attracted scientific attention, not only to describe behavioral patterns but also to evaluate how environmental, nutritional, and physiological conditions influence this state. Since the first electrophysiological descriptions of sleep in cattle [5], research activity has remained relatively limited and predominantly focused on dairy systems under confinement. Consequently, the available literature represents a limited range of cattle populations, management systems, and environmental conditions. Early studies were constrained by methodological limitations, including invasive procedures, intensive animal handling, and high operational costs, which restricted broader application in production environments [12,13].

Advances in noninvasive polysomnography using surface electroencephalography (EEG), electromyography (EMG), and electrooculography (EOG) recordings have improved the feasibility of sleep assessment in freely moving cattle [12]. These developments enabled more refined characterization of sleep in experimental and production contexts and supported the validation of complementary approaches for monitoring vigilance states. Despite these methodological improvements, sleep assessment in cattle remains largely restricted to controlled experimental conditions.

Beyond methodology, sleep is linked to physiological systems relevant to cattle function. In humans, sleep duration influences metabolic hormones such as leptin and ghrelin, which regulate appetite and energy balance [7]. In cattle, leptin is associated with feed intake regulation and body composition traits, including marbling, while ghrelin stimulates appetite and is linked to feed intake dynamics [14,15,16,17]. However, the role of leptin in regulating feed intake in ruminants remains debated. For example, serum leptin explained only 0.54% of the variation in DMI of finishing beef steers after accounting for body size and fatness, whereas stronger associations were observed with measures of adiposity [15]. These findings suggest that circulating leptin may be more closely associated with long-term energy status and body fatness than with variation in feed intake itself. Whether sleep-related changes in these endocrine pathways have functional consequences in cattle remains unknown.

Sleep regulatory networks and the immune system have been extensively described in mammals [8]. Sleep disruption can enhance pro-inflammatory signaling, including cytokines such as IL-6 and TNF-α, thereby impairing both innate and adaptive immune responses [9]. Conversely, immune activation can also modify sleep, with pro-inflammatory cytokines increasing NREM sleep in experimental animal models [9]. Thus, increased sleep should not necessarily indicate improved physiological state or welfare. Cattle under production conditions may experience nutritional, environmental, and microbial challenges associated with inflammatory responses and increased physiological demands [10,11]. However, direct evidence linking sleep variation with immune function in cattle remains limited.

Collectively, evidence from mammals indicates that sleep is associated with endocrine regulation, immune function, and physiological homeostasis, providing plausible biological pathways for functional consequences of sleep variation in cattle. However, direct causal relationships between variation in sleep and health, welfare, or productive performance have not been established in cattle. Sleep should therefore not currently be considered a validated indicator of these outcomes. A similar interpretive limitation has been recognized for lying behavior in dairy cattle: although reduced lying time may occur under unfavorable conditions, increased lying time does not necessarily indicate better welfare because lying behavior is also influenced by health status, physiological state, and environmental conditions [18]. Changes in sleep may reflect responses to environmental or physiological challenges, but adaptation of sleep timing or architecture should not necessarily be interpreted as evidence of good welfare. Determining whether such changes represent successful adaptation, biological cost, or both will require concurrent assessment of sleep and established physiological and behavioral indicators of welfare and function. To interpret sleep as a biological variable in cattle, we first need to define its physiological organization, including its fundamental REM and NREM architecture.

## 4. REM and NREM Sleep

In mammals, sleep is organized into REM and NREM states, with NREM further divided into lighter and deeper substages culminating in slow-wave sleep. These states reflect distinct neurophysiological configurations and differ fundamentally in cortical activation patterns, muscle tone, and autonomic regulation [1,19,20].

Sleep differs from other reversible states of reduced consciousness by its responsiveness to external stimuli and rapid reversibility, reflecting an active and regulated brain state rather than passive inactivity [1]. Across mammals, REM sleep typically involves cortical activation and muscle atonia, whereas NREM sleep is characterized by synchronized cortical activity and reduced metabolic demand.

During NREM sleep, parasympathetic activity predominates, reflected in reduced heart rate and increased digestive activity. REM sleep shows a relative shift toward sympathetic activity, with transient cardiovascular activation and reduced gastrointestinal activity [21,22]. Central autonomic networks coordinate these autonomic changes by integrating neural, endocrine, and behavioral outputs across vigilance states.

Electrophysiologically, NREM sleep is defined by synchronized cortical activity with high-amplitude, low-frequency oscillations, reflecting widespread neuronal coherence and reduced cortical information processing. In contrast, REM sleep shows desynchronized, low-amplitude mixed-frequency activity that resembles wakefulness at the cortical level but occurs under muscular atonia and behavioral quiescence [6,23,24,25]. This dissociation between cortical activation and motor suppression defines REM sleep.

In cattle, REM and NREM sleep were first systematically described using electrophysiological recordings by Merrick and Scharp [5] and later formally classified by Ruckebusch [6]. Across studies, cattle spend the majority of their total sleep time in NREM, with REM accounting for a smaller fraction of total sleep (Table 1). Table 1 includes both baseline (undisturbed) recordings and experimental conditions involving sleep or lying deprivation, allowing comparison between unmanipulated and experimentally altered sleep patterns.

Early studies reported higher absolute sleep durations than several subsequent studies [6,26]. Because animals with implanted electrodes were recorded over extended periods, acute effects of surgery or perioperative sedation are unlikely to explain these higher values fully. More recent studies using noninvasive PSG have generally reported lower sleep estimates [12,25,27,28], although the magnitude and causes of this difference remain uncertain. Differences in electrode configuration, scoring criteria, signal quality, experimental conditions, and how researchers handle periods affected by rumination may contribute to variation across studies. Thus, the discrepancy should not be interpreted simply as overestimation by invasive methods or greater accuracy of noninvasive recordings.

In adult dairy cows, recent studies using noninvasive PSG have generally reported total sleep durations of approximately 1.2–2.3 h per 24 h [25,27,28]. These values should not, however, be interpreted as a species-wide estimate. In 3-month-old calves, Hänninen et al. [12] reported that sleep accounted for approximately 25% of the observation period (14.0% NREM and 11.2% REM sleep), indicating that age or developmental stage may substantially influence sleep duration and architecture. Because a further 40% of observations were classified as rumination, during which the vigilance state could not be determined, and only about 15 h of each 20-h recording were readable, the corresponding daily duration depends on the time base: approximately 3.8 h of sleep in the readable data, 5.0 h when scaled to the 20-h recording window, and 6.0 h when extrapolated to 24 h (Table 1). Moreover, most available physiological recordings were obtained under controlled experimental conditions, limiting inference about sleep duration in other management and environmental contexts. Noninvasive recordings also remain susceptible to signal artifacts and uncertain classification of vigilance states, particularly during rumination [12,29,30]. Consequently, researchers must consider both biological and methodological sources of variation when interpreting reported sleep durations.

REM sleep in cattle appears particularly sensitive to experimental perturbations. Preventing cows from lying down for 22 h per day almost abolished REM sleep [26], and 24 h of lying deprivation reduced NREM sleep from 1.1 to 0.3 h and tended to reduce REM sleep [25]. In contrast, gentle handling intended to prevent sleep while allowing cows to lie down did not change the duration of any vigilance state [25], indicating that sleep itself is difficult to manipulate experimentally in cattle. Physiological state also modulates sleep architecture: periparturient and lactation phases are associated with changes in total sleep duration and stage distribution [24,28]. After several weeks of restricted recumbency, NREM and REM sleep rebounded to almost twice their usual values for four days [26], supporting a homeostatic regulatory drive.

These findings indicate that experimental and physiological conditions influence sleep in cattle, although it is not always clear how much reported differences reflect biological responses versus methodological factors. Importantly, behavioral signs such as recumbency, reduced movement, and characteristic head postures may indicate sleep-like behavior but do not necessarily correspond to physiologically defined sleep. This distinction matters because cattle also exhibit drowsing, an intermediate vigilance state between wakefulness and NREM sleep that can share behavioral features with both states. The following section discusses the physiological and behavioral characteristics of drowsing.

## 5. Drowsing: The Intermediate State Between Wakefulness and NREM Sleep

Drowsing has been described in cattle as an intermediate vigilance state between alert wakefulness and NREM sleep, characterized by reduced responsiveness and partial disengagement from environmental stimuli [6]. Within the sleep–wake continuum, this state is considered transitional, with spontaneous progression either toward full alert wakefulness or into NREM sleep, reflecting its unstable neurobehavioral expression.

Physiologically, drowsing is characterized by electrophysiological features intermediate between wakefulness and NREM sleep. Compared with wakefulness, EEG activity becomes more synchronized and muscle activity is reduced, whereas the EEG remains less regular than during NREM sleep and contains a mixture of low- and high-frequency activity [6,25,27]. The operational criteria used to separate drowsing from adjacent states, however, differ between studies (Table 2). Ruckebusch [6] defined drowsing as a mixture of low-voltage fast and high-voltage slow ECoG activity with a small decrease in muscle tone, heart rate and respiratory rate, partial closure of the eyelids and a low arousal threshold to auditory stimulation, which increased approximately tenfold in slow-wave sleep. Ternman et al. [29] introduced a spectral criterion, scoring an epoch as drowsing when the EEG was synchronized and power in the 10–30 Hz band was between 0.2 and 1.0 µV^2^/Hz, and as NREM sleep when this power was below 0.2 µV^2^/Hz and EMG activity was reduced; K-complexes were frequent during drowsing. Kull et al. [25] applied a qualitative definition (a less variable, usually synchronized EEG with some 10–30 Hz activity), Hänninen et al. [12] could not distinguish drowsing from NREM sleep in calves, and Hunter et al. [23] did not score drowsing but subdivided NREM sleep into stages N1–N3. Drowsing is therefore separated from quiet wakefulness mainly by EEG synchronization and reduced EMG activity, and from NREM sleep by a spectral threshold that has been made explicit by only one research group and has not been validated against an independent criterion such as arousal threshold. Behaviorally, drowsing may be accompanied by reduced vigilance and motor activity, but these signs are not specific and may also occur during quiet wakefulness, rumination, or light sleep. Behavioral observations alone therefore cannot reliably distinguish drowsing from adjacent vigilance states in adult cattle. In adult cows, drowsing identified by PSG occurred mostly while cows were lying with the head lifted and still, the posture also used to indicate NREM sleep [31].

**Figure 1:**
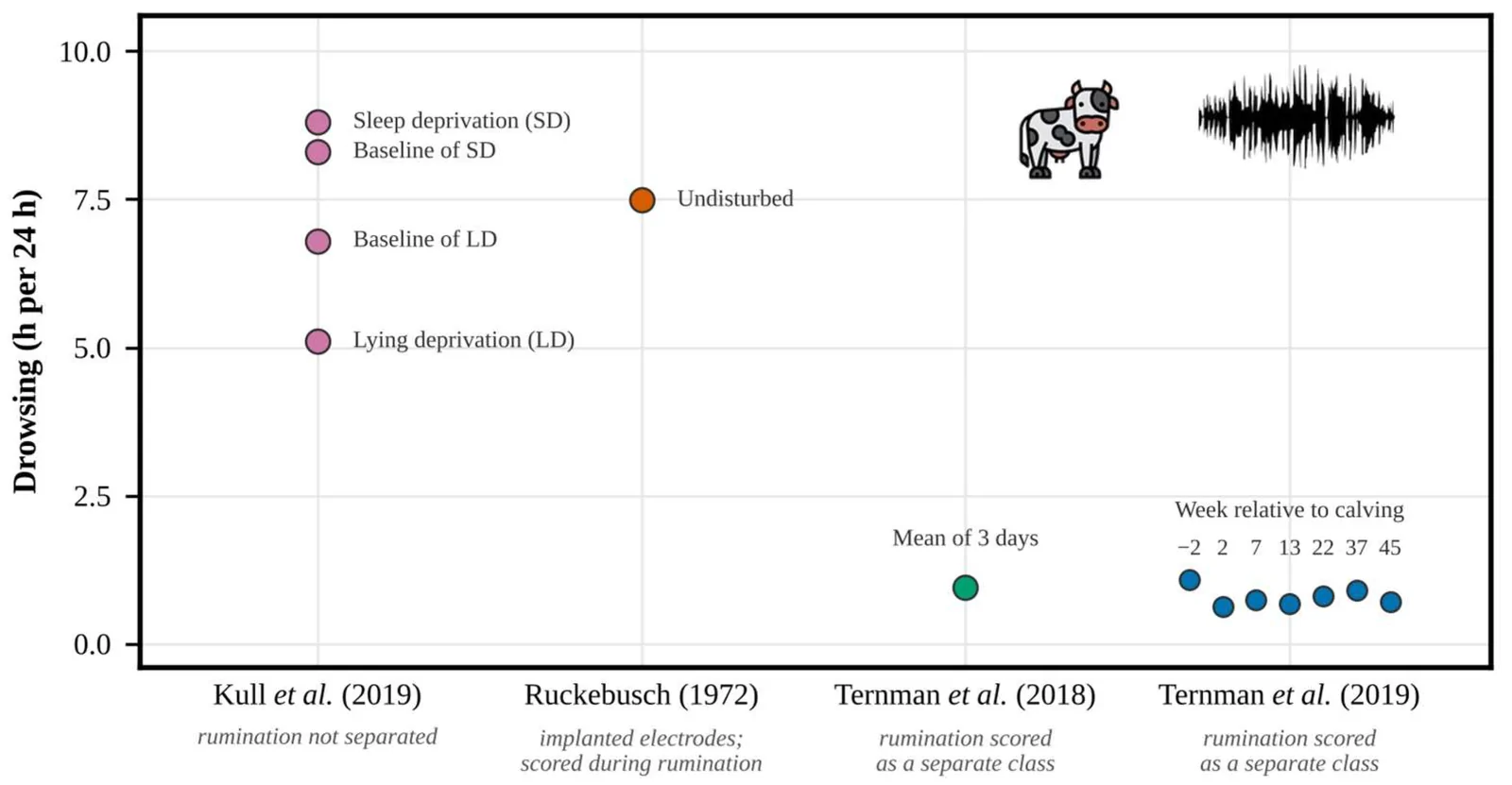
Daily duration of drowsing (DR, h per 24 h) in electrophysiological studies of cattle. Each point represents one experimental condition: the baseline and treatment days of the sleep-deprivation (SD) and lying-deprivation (LD) experiments of Kull et al. [25]; undisturbed cows in Ruckebusch [6]; the mean of three consecutive recording days in Ternman et al. [27]; and the seven recordings from 2 weeks before to 45 weeks after calving in Ternman et al. [28]. Colors indicate studies: Kull et al. [25] in purple, Ruckebusch [6] in orange, Ternman et al. [27] in green and Ternman et al. [28] in blue. The handling of rumination in each study is given below the study name; numerical values are given in Table 1.

Quantification of drowsing in cattle shows substantial heterogeneity across the literature (Table 1 and Figure 1). Ruckebusch [6] reported that drowsing occupied on average 31% of the 24-h cycle (7 h 29 min) in three cows, whereas Ternman et al. reported 38–65 min per day, or approximately 2.5–4.5% of total time, using surface electrodes [27,28]. Kull et al. [25], also using surface electrodes, reported 6.8–8.3 h per day under baseline conditions, values comparable to those of Ruckebusch [6] rather than intermediate. Two considerations reconcile these figures with the much shorter total sleep times reported in the same studies. First, drowsing is classified as a wakeful state and is not included in total sleep time in any of these studies. Second, the studies differed in how they treated rumination. In the studies of Ternman et al., rumination was scored as a separate category because chewing artifacts prevented classification of the underlying vigilance state; it occupied 7.6–8.8 h per day, and drowsing or NREM sleep occurring during rumination could therefore not be counted [27,28]. Kull et al. [25] did not score rumination separately, so that wakefulness, drowsing, NREM and REM sleep summed to 24 h, and Ruckebusch [6], using implanted electrodes, was able to classify the ECoG during rumination and observed that drowsing frequently coincided with it. Much of the variation in reported drowsing, and part of that in NREM sleep, is therefore likely to reflect whether time spent ruminating was excluded, absorbed into wakefulness and drowsing, or classified physiologically, rather than biological differences between animals. Figure 1 visually summarizes these differences.

Figure 1 illustrates the wide dispersion in reported drowsing durations across experimental conditions and studies, ranging from minutes to several hours. Differences in experimental design, recording characteristics, physiological state, operational criteria for distinguishing drowsing from quiet wakefulness and NREM sleep and, in particular, the handling of rumination may contribute to this variation. Consequently, direct comparisons of drowsing duration across studies require caution.

Experimental and physiological conditions may also influence drowsing. Preventing recumbency for 22 h per day approximately halved drowsing [26], and 24 h of lying deprivation tended to reduce it (from 6.8 to 5.1 h per day), whereas gentle handling intended to prevent sleep did not change it [25]; drowsing was also shorter 2 and 13 weeks after calving than 2 weeks before calving [28]. However, it remains unclear how much of these differences reflect changes specifically in drowsing rather than redistribution among adjacent vigilance states.

A particularly unresolved aspect of drowsing in cattle is its relationship with rumination. Early electrophysiological observations indicated that drowsing frequently occurred during rumination [6], raising the possibility that rumination may coincide with reduced vigilance. However, whether cattle can enter physiologically defined NREM sleep while actively ruminating remains uncertain. Evidence from another ruminant species suggests that this question may have a physiological basis. In reindeer, rumination was associated with NREM-like EEG features, including slow waves and sleep spindles, and sleep pressure decreased during rumination at a rate comparable to that observed during NREM sleep [32]. Moreover, reindeer that ruminated more spent less time in NREM sleep [32]. These observations, obtained in four animals of a cervid species living under extreme seasonal conditions, are indirect evidence and cannot establish a sleep-related role of rumination in cattle. Such an extrapolation would require three mechanisms to be conserved: cortical synchronization during rumination, homeostatic regulation of sleep reflected in NREM slow-wave activity, and dissipation of sleep pressure during rumination. In cattle, only the first two have some support: drowsing with delta activity, and occasionally slow-wave sleep, was recorded during rumination with implanted electrodes [6], and prolonged prevention of recumbency was followed by a rebound of NREM and REM sleep [26]. Whether slow-wave activity declines during rumination, or whether cows that ruminate more need less NREM sleep, has not been tested. A behavioral observation is consistent with, but does not demonstrate, such a trade-off: cows fed a concentrate-rich diet ruminated approximately 260 min per day less and spent approximately 27 min per day more in sleep posture than when fed roughage [33]. This question is difficult to resolve with noninvasive polysomnography because rhythmic masticatory muscle activity produces substantial artifacts in EEG and EMG signals, complicating reliable classification of vigilance states during rumination [12,29,30]. Consequently, rumination periods may represent an important source of unclassified or potentially misclassified time in estimates of sleep and drowsiness in adult cattle. Differences in how researchers identify and treat rumination periods may also contribute to discrepancies in drowsing estimates between early implanted-electrode studies and more recent surface-recording studies. Thus, the relationship between rumination, drowsing, and sleep remains both a biological question and a methodological limitation in cattle sleep research.

Overall, current evidence indicates that drowsing represents a distinct but incompletely characterized component of the cattle sleep–wake continuum. Its overlap with adjacent vigilance states and rumination remains a major source of uncertainty in both physiological and behavioral classification.

## 6. Challenges to Measuring Sleep in Cattle

The interaction between physiological state complexity and methodological limitations inherently constrains sleep assessment in cattle. Across available approaches, including PSG, behavioral observation, and sensor-based systems, sleep is inferred from characteristic physiological or behavioral signatures rather than measured as a single, directly observable variable. PSG provides the physiological reference for sleep-stage classification, whereas behavioral and sensor-based estimates require validation against physiologically defined vigilance states.

Polysomnography remains the reference standard for sleep classification in cattle, but its application introduces important experimental constraints. Early studies relied on invasive electrode implantation under sedation [5,6,24,26], although animals were subsequently recorded over extended periods, making it difficult to establish persistent effects of the implantation procedure or perioperative sedation. Contemporary noninvasive protocols using surface electrodes reduce surgical invasiveness, but the degree of animal preparation and restraint varies among recording systems. Regardless of configuration, maintaining electrode contact and signal quality in freely moving cattle remains a practical challenge and may require controlled experimental conditions, potentially limiting ecological validity [13,30].

A second major limitation is physiological signal contamination, particularly from rumination. Adult cattle typically spend approximately seven hours per day ruminating, although this varies with diet composition, fiber content, and intake level [34]. Rumination generates intense jaw and facial muscle activity, introducing substantial artifacts into EEG and EMG recordings. These artifacts can distort or mask electrophysiological signatures used to define sleep stages, particularly NREM sleep, creating physiological noise intrinsic to ruminant biology rather than methodological error [12,29,30]. The resulting difficulty in reliably classifying vigilance states during rumination may create substantial periods of uncertainty in noninvasive sleep recordings. Consequently, estimates of total sleep and, particularly, drowsing and NREM sleep should be interpreted with caution when rumination periods cannot be physiologically classified.

Behavioral assessment adds another layer of ambiguity because of intermediate vigilance states, such as drowsing. Drowsing shares behavioral features with both wakefulness and NREM sleep, including recumbency and reduced responsiveness, but lacks a clear physiological boundary when assessed without concurrent electrophysiological data. As a result, behavioral methods exhibit limited specificity, particularly in distinguishing quiet wakefulness from drowsing and physiologically defined sleep [31].

Emerging sensor-based approaches, including accelerometers and heart rate monitoring, have been proposed for scalable monitoring, but none has yet been validated against PSG under production conditions. Their limitations fall into systematic errors and physiological confounding. Approaches based on immobility and posture are prone to a structural classification bias rather than random noise: in calves, an accelerometer-based classifier assigned 45% of epochs in which calves lay awake to NREM sleep posture and overestimated NREM time [35]. This evidence comes from young calves, a neck-mounted sensor and a single algorithm, and was obtained against video-scored postures rather than PSG; the magnitude of the bias in adult cattle, with other sensor positions or algorithms, is unknown. In contrast, heart rate–based approaches are primarily affected by physiological confounders, including autonomic modulation during rumination and other non-sleep-related states, reducing specificity for sleep staging [30]. Machine-learning approaches can integrate several of these signals, but their accuracy is bounded by the quality of the physiological reference used for training and validation (Section 7.2).

These limitations do not preclude physiologically informed sleep assessment in cattle, but the reliability of sleep estimates depends on the method used, the vigilance state being classified, signal quality, how unclassifiable periods are handled, and the extent of physiological validation.

## 7. Methods to Measure Sleep and Its Stages

### 7.1. Behavioral Observation and Posture

Sleep-related states in cattle can be assessed using methodologies that differ substantially in physiological resolution, cost, labor requirements, and applicability under commercial conditions, ranging from behavioral assessment of sleep-like states to physiological sleep-stage classification using PSG.

Behavioral observation is the simplest and most widely applicable approach for identifying sleep-like states under field conditions. Because it does not require specialized equipment or animal preparation, it allows researchers to evaluate large numbers of animals under commercial production settings. However, behavioral methods do not measure physiological sleep directly and instead rely on postural and activity-based proxies whose correspondence with physiologically defined vigilance states varies with age and sleep stage.

In calves, body posture provides a relatively reliable estimate of sleep stages [12,35]. An upright and motionless head is commonly associated with NREM sleep, whereas a relaxed neck posture with the head resting against the body or on the ground, known as sleep-like posture (SLP), is strongly associated with REM sleep [12,35]. In 3-month-old calves, Hänninen et al. [12] found close agreement between behavioral and electrophysiological estimates of total sleep duration, although individual postures were substantially less sensitive for identifying NREM and REM sleep. Thus, behavioral observation may estimate overall sleep occurrence more accurately than individual sleep stages. SLP decreases markedly during early development and stabilizes around 12 months of age, suggesting a relationship with neurological maturation [36].

In adult cattle, however, postural indicators become considerably less reliable. Studies combining behavioral observations with PSG have demonstrated that classical sleep postures frequently overestimate physiologically defined sleep duration [23,31]. The principal source of error is the overlap among quiet wakefulness, drowsing, and sleep, as adult cows may exhibit similar postures during these states. Likewise, the characteristic tucked-head posture traditionally associated with REM sleep may also occur during wakefulness or drowsing, reducing its predictive value [23,31]. Consequently, posture alone cannot reliably distinguish vigilance states in adult cattle.

Despite these limitations, resting postures may provide useful information on resting behavior under field conditions, and their frequency has been associated with housing conditions, feeding management, and environmental quality [33,37,38]. Resting behavior, including lying or inactivity, should not be interpreted as evidence of sleep. Specific postures or behavioral patterns explicitly proposed as indicators of sleep may be described operationally as sleep-like or sleep-related behaviors; however, because their correspondence with physiological sleep is limited, they should not be interpreted as direct measures of sleep unless validated against concurrent physiological recordings.

### 7.2. Accelerometers and Wearable Monitoring Approaches

Wearable accelerometers are used in humans to estimate physical activity and sleep–wake cycles [39,40] and in cattle to detect behavioral changes associated with disease. However, human consumer wearables commonly integrate movement with additional physiological signals, such as heart rate, whereas accelerometry alone has limited ability to distinguish sleep from motionless wakefulness. Direct extrapolation to cattle is further limited by species differences in sleep architecture and daily organization, including the more polyphasic distribution of sleep in cattle. Accelerometers measure movement along one or more spatial axes, and algorithms classify the resulting signals into behavioral states [39].

In cattle, three-dimensional accelerometers attached to the head, neck, or collar have emerged as a practical approach for automatically detecting sleep-related postures and body positions, reducing the need for continuous visual observation [35,41,42]. Their main potential advantage is scalability; however, none of the accelerometer-based approaches developed for cattle has been validated against PSG, and agreement has been assessed only against video or direct behavioral observation.

Nevertheless, accelerometers only measure physical behavior rather than sleep itself. Consequently, they share many limitations with visual observation, particularly the limited ability to distinguish among quiet wakefulness, drowsing, and physiologically defined sleep [42]. Periods of behavioral inactivity may therefore be classified as sleep-like states even when physiological evidence of sleep is absent, potentially overestimating sleep duration. In calves, a neck-mounted accelerometer with a support vector machine classifier estimated total time in sleep postures well (90% of observed time) but classified 45% of epochs in which calves lay awake as NREM sleep posture, overestimating NREM and underestimating REM time [35]. In adult cows, an accelerometer classifier distinguished wake from sleep-like behavior with more than 92% accuracy, but only after segments with uncertain behavior or transitions had been removed and against behavioral observation rather than PSG [42], and occipital accelerometers detected the tucked sleep posture in close agreement with video [43], a posture that itself identifies REM sleep inconsistently [23].

With respect to the three states most easily confused in practice, current evidence allows only partial conclusions. Standing and lying can be separated reliably by accelerometers (96% of lying time correctly predicted in calves [35]), and because adult cattle rarely enter NREM sleep while standing unless recumbency is prevented [26], excluding standing epochs is unlikely to bias sleep estimates substantially under normal conditions. Rumination can be detected from jaw movements in close agreement with electrophysiologically identified rumination (sensitivity 96% and specificity 94% in calves [12]), but the vigilance state during rumination cannot be determined with surface PSG, so no sensor can currently be validated for that time. The critical unresolved distinction is between lying quiet wakefulness or drowsing and NREM sleep, which share posture and immobility [31,35]. No accelerometer-based method has been shown to make this distinction against a physiological reference.

As summarized in Table 3, the available literature is dominated by studies of dairy cows maintained under confined conditions, frequently with small sample sizes. Of the 22 primary studies, 14 were based on electrophysiological recordings, four with implanted and ten with surface electrodes, whereas eight relied exclusively on postures recorded by video or accelerometers. Physiologically validated measurements across diverse animal categories, management systems, and environmental conditions remain scarce. The table also illustrates that studies based solely on behavioral or movement-based measures should be distinguished from those using physiological recordings to classify sleep states. This limited diversity may also constrain the generalizability of behavioral prediction models, because the relationship between movement or posture and physiological sleep may vary across housing, management, and environmental conditions. Models should therefore be validated in independent populations and production systems before their performance is assumed to transfer across conditions.

Machine-learning algorithms can be applied to signals from different monitoring systems rather than serving as an independent sleep-measurement method. Recent studies have used these algorithms to integrate multiple physiological signals and automate vigilance-state classification. Combining information from EMG, HR, and HRV enabled classification of wakefulness, light (N1/N2) and deep (N3) NREM sleep, and REM sleep with an overall accuracy of approximately 82% in 12 dairy cows [13]. However, this approach excluded standing and rumination periods before classification [13]. In addition, 72% of the remaining epochs were wakefulness, so that the per-class accuracies reported for N3 and REM sleep (95.3% and 95.8%) are similar to those that would be obtained by never predicting these states (95.5% and 95.2%), and cross-validation was performed on randomly split epochs, so that epochs from the same cows were used for both training and testing [13]. Heart rate and heart rate variability do differ between sleep stages in cows, but mean heart rate also differed markedly between the two cohorts studied, which differed in breed, pregnancy status and season [45]. Thus, the reported accuracy reflects classification within a preselected subset of behavioral states rather than performance across cattle’s full behavioral repertoire. This limitation is particularly relevant for field application because rumination occupies a substantial proportion of the daily time budget. Moreover, the performance of machine-learning models ultimately depends on the quality of the physiological reference used for training and validation. Although promising, these approaches therefore require validation using datasets that retain the major behavioral states and adequately represent conditions encountered during continuous monitoring. Claims of field applicability are therefore premature. Before indirect methods are used to quantify sleep under production conditions, they should be validated against PSG at the animal level, for example by leave-one-animal-out cross-validation or in independent cohorts, retaining standing, lying wakefulness, drowsing and rumination as explicit classes and reporting class-specific sensitivity, precision and agreement (Table 4).

### 7.3. Gold Standard: Polysomnography (PSG)

Despite advances in behavioral and wearable monitoring approaches, PSG remains the reference standard for physiological sleep assessment in cattle. For sleep-stage classification in the cattle studies reviewed here, PSG principally integrates EEG to assess cortical activity, EOG to detect eye movements, and EMG to assess muscle activity and tone. Together, these signals provide the physiological basis for distinguishing wakefulness, drowsing, NREM sleep, and REM sleep, although classification may be limited when signal quality is compromised.

Modern PSG developed after Berger introduced EEG in humans [46] and later integrated it with EOG and EMG for standardized sleep-stage classification [47,48,49].

In cattle, electrode placement and animal preparation vary by recording system and electrode configuration. In several noninvasive PSG protocols, researchers clip hair from selected regions of the head and neck and clean the skin to improve electrode contact and signal quality [12,27,31]. Surface electrodes may then be secured using conductive media and adhesive materials. Common configurations include EEG electrodes positioned on the forehead, EOG electrodes around the eyes, and EMG electrodes on the neck musculature [12,13,23,25,27].

**Figure 2:**
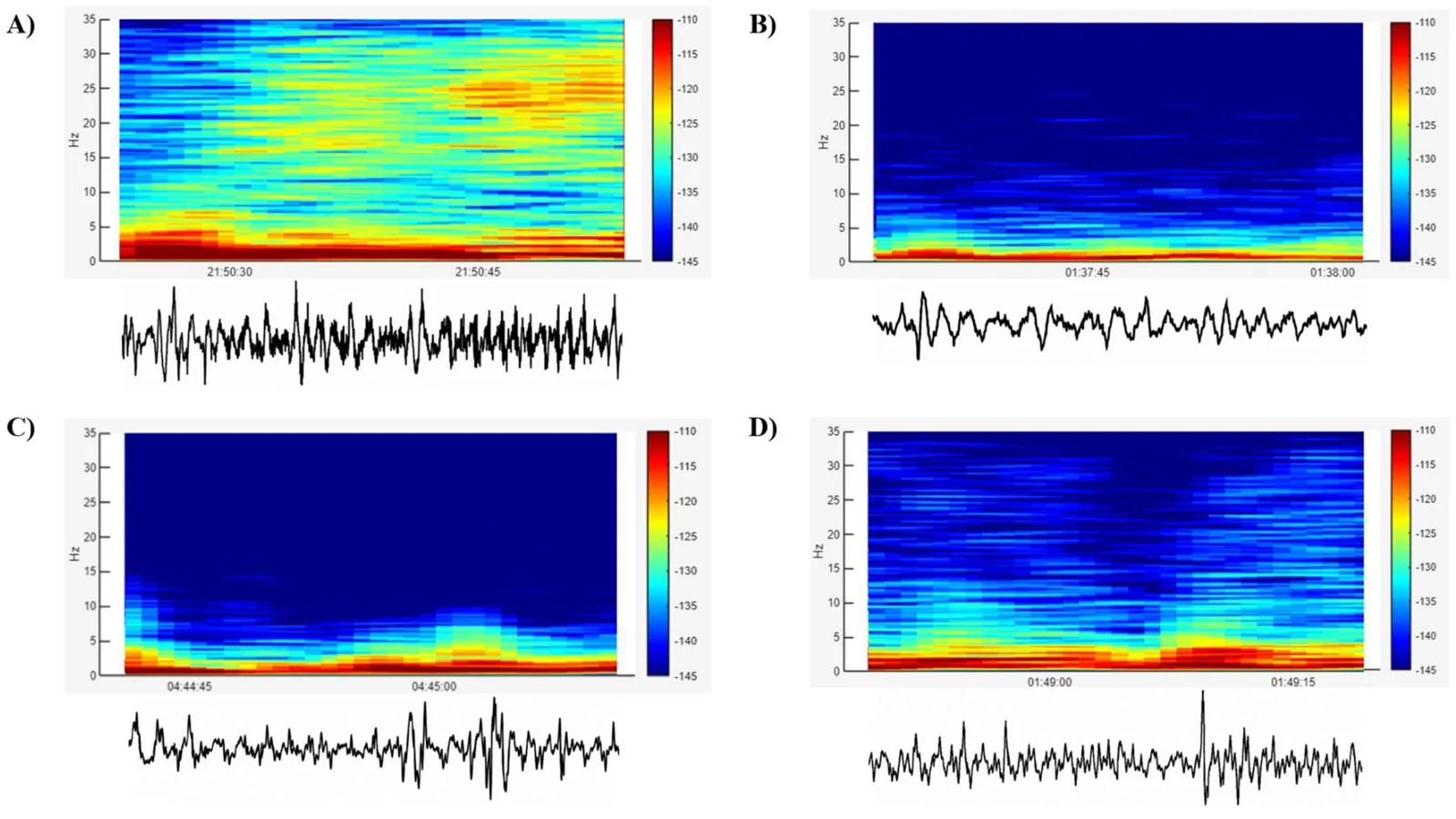
Representative EEG spectrograms and corresponding waveforms recorded during four vigilance states in beef cattle (30-s epochs): (**A**) wakefulness, (**B**) drowsing, (**C**) NREM sleep, and (**D**) REM sleep. Wakefulness is characterized by desynchronized EEG activity with greater power distributed across a broad frequency range. Drowsing represents an intermediate vigilance state between alert wakefulness and NREM sleep, showing increased low-frequency activity without the synchronized pattern typical of NREM sleep. NREM sleep exhibits synchronized EEG activity with predominant power in the delta frequency range (approximately 1–4 Hz). REM sleep is characterized by a return to a desynchronized EEG pattern with reduced delta activity compared with NREM sleep. The recordings were obtained in a pilot study conducted by our group, the results of which were previously reported in abstract form [50]; the EEG recordings shown here were not included in that publication.

After acquisition, researchers divide physiological recordings into standardized epochs, typically 30 s long. Trained scorers evaluate each epoch based on combinations of cortical activity, eye movements, and muscle tone, enabling classification of alert wakefulness, drowsing, NREM sleep, and REM sleep [23]. However, epochs affected by substantial artifacts may remain difficult or impossible to classify reliably, particularly when muscle activity associated with rumination contaminates physiological signals. Reporting of scoring procedures is incomplete (Supplementary Table S2). Inter-scorer agreement was reported only by Kull et al. [25], who required four scorers to reach at least 90% agreement with an expert on a pre-scored recording, and intra-scorer agreement only by Hunter et al. (89.4%; κ = 0.83), with agreement falling to 32% for the distinction between N1 and N2 sleep [13,23]. Scoring was performed by a single person in several studies [12,13,23], blinding of the scorer to behavior was reported in one [31], and the proportion of unreadable data, when reported, ranged from 3.6% of recording time [29] to approximately one quarter, with a further 40% of readable observations classified as rumination [12]. In the studies of Ternman et al., rumination, during which no vigilance state could be assigned, accounted for 7.6–8.8 h per day [27,28]. Electrophysiological reference labels in cattle are therefore reliable mainly for non-ruminating epochs, and REM sleep, during which cattle do not ruminate, is the state whose daily duration can be estimated with most confidence [28]. Figure 2 provides illustrative examples of EEG spectrograms and waveforms for wakefulness, drowsing, NREM sleep, and REM sleep obtained from beef cattle in a pilot study conducted by our group, the results of which were previously reported in abstract form [50]. The recordings are shown to illustrate the electrophysiological features used for sleep-stage classification and are not included as primary evidence in the synthesis of this review.

Despite its status as the reference method, PSG has significant limitations for field research: depending on the recording system, it may require animal habituation, technical expertise, extensive data processing, and specialized equipment, and noninvasive recordings remain susceptible to the artifacts described in Section 6. Importantly, validation of alternative approaches against PSG is meaningful only when the physiological reference itself can be classified with sufficient confidence. Under current methods, validation is therefore conditional: agreement statistics apply to the non-ruminating, and usually lying, epochs that could be scored, and should be reported and interpreted as such. Two steps would make such validation more informative: reporting unscorable epochs as a separate class, so that the proportion of time for which an alternative method cannot be evaluated is explicit, and testing whether conclusions change when these epochs are treated differently, as illustrated by the dependence of drowsing estimates on the handling of rumination (Table 1). Therefore, improving signal quality and establishing transparent criteria for handling artifact-contaminated and unclassifiable epochs are prerequisites for robust validation of alternative approaches. Table 5 summarizes the principal strengths and limitations of the available assessment approaches.

## 8. Integrative Synthesis: Interpreting Sleep in Cattle

Current evidence supports physiologically distinguishable vigilance states in cattle, including wakefulness, drowsing, NREM sleep, and REM sleep, but confidence in their quantification differs substantially across states and methodologies. Electrophysiological recordings can distinguish REM and NREM sleep, whereas drowsing remains less consistently defined and overlaps with quiet wakefulness, NREM sleep, and rumination [6,12,25,27]. Consequently, disagreement among studies may reflect not only biological variation but also differences in how vigilance states are operationally defined and classified.

This methodological dependence is evident in the large variation in reported sleep and drowsing durations, much of which is likely to reflect the classification criteria and the handling of rumination rather than biological differences between animals (Section 4 and Section 5; Table 1). Rumination may also be more than a source of artifact, but whether it contributes to sleep regulation in cattle, as suggested for reindeer [32], has not been tested (Section 5). Thus, some apparent biological variation in cattle sleep may reflect methodological uncertainty rather than true differences in sleep expression or architecture.

These limitations also establish a hierarchy for interpreting available measurements. Behavioral observations and accelerometry can characterize resting or sleep-like states at scale but cannot independently establish physiological sleep in adult cattle. PSG provides the principal physiological reference currently available for differentiating vigilance states, although it is itself susceptible to artifacts and uncertain classification during specific behaviors, particularly rumination. The inclusion of standing, lying wakefulness, drowsing, rumination, and other potentially confounding states in validation datasets does not make these behaviors reliable indicators of sleep; rather, it allows the ability of automated methods to distinguish physiological sleep from competing behavioral states to be tested. Their performance should therefore be evaluated against physiologically defined reference states, while explicitly accounting for epochs in which the physiological reference itself is uncertain or unclassifiable.

Table 6 summarizes the factors whose effects on sleep or sleep-like behavior have been tested in cattle, with the direction and magnitude of the reported effects and the type of evidence. Apart from the day–night distribution of sleep, which consistently favors the night [6,27,28], only two factors have been examined with electrophysiological methods in more than one study, and in both the direction of the effect was consistent: preventing recumbency reduced REM sleep [26], with a similar tendency in [25], and reduced NREM sleep [25], and sleep was shorter around calving and in early lactation than in late gestation [24,28]. Only the first is supported by experimental manipulation; the second derives from observational designs in which parturition, lactation and management changes are confounded. All other factors, diet [33], cleanliness of bedding [37], outdoor access [38], relocation [41] and age [36], were evaluated in single studies of 5–15 animals using posture-based proxies, so their effects on physiological sleep remain unknown. Evidence linking sleep loss to health or production is correspondingly limited. Lying deprivation, which reduced NREM sleep, was followed by lower milk yield on the next two days [25] and by increased expression of pro-inflammatory cytokines (TNF, IL1B and IL6) in blood leukocytes [44], but because lying and sleep were lost together these effects cannot be attributed to sleep loss alone, and gentle handling that failed to reduce sleep had no such effects [25,44]. Outdoor access increased T-cell proliferation in tethered cows, but sleep was inferred only from posture [38].

A useful framework therefore considers cattle sleep assessment along two complementary dimensions: the biological state being investigated and the certainty with which that state can be identified. Changes in sleep duration, stage distribution, fragmentation, or timing may reflect responses to environmental, physiological, or management conditions, but these responses should not automatically be interpreted as beneficial adaptation or compromised welfare. This need for contextual interpretation is consistent with evidence on lying behavior in dairy cattle, where both reduced and increased durations may occur under biologically relevant conditions, and no single daily threshold provides an unequivocal measure of welfare [18]. Likewise, resting behavior should not be treated as synonymous with physiological sleep. The welfare relevance of altered sleep can only be established by determining whether such changes are consistently associated with independent indicators of affective state, physiological function, health, or behavioral need. Establishing the functional relevance of sleep will therefore require studies that simultaneously characterize vigilance states, relevant behavioral contexts such as rumination, and independent indicators of physiological function and welfare. Within this framework, the central question is no longer how long cattle sleep, but how reliably different vigilance states can be identified, which biological or environmental factors alter them, and whether these alterations have measurable functional or welfare consequences.

## 9. Future Perspectives for Sleep Research in Cattle

Sleep in cattle also has a temporal dimension that warrants further investigation. Cattle exhibit polyphasic sleep distributed across the 24-h cycle, with sleep and resting behavior occurring predominantly at night and REM sleep episodes tending to be longer during this period [27]. These day–night differences were recorded under fixed periods of artificial light and fixed milking and feeding times [27,28], so they describe the daily distribution of sleep under these schedules rather than demonstrating endogenous circadian regulation, which would require recordings under constant conditions or after experimental shifts in these cues. However, sleep timing is flexible: following nighttime lying deprivation, cows can shift REM sleep expression to daytime periods during recovery [26]. These observations suggest an interaction between daily temporal organization and homeostatic sleep pressure, but the relative contribution of circadian and homeostatic regulation to sleep in cattle remains poorly understood. Future studies should determine how management-related temporal cues, such as photoperiod and feeding or milking schedules, interact with sleep timing and whether disruption of these patterns has measurable consequences for cattle physiology and welfare [51,52].

Research on sleep in cattle has advanced considerably over the last two decades, particularly following the development of noninvasive polysomnography. Nevertheless, the current body of knowledge remains relatively small and concentrated within a narrow range of production systems and animal categories. Most studies have been conducted with dairy cows housed under confined conditions, while other cattle populations and production environments remain poorly represented. Consequently, the extent to which current findings generalize across breeds, animal categories, management systems, and environmental conditions remains uncertain. Future studies should therefore broaden the biological and production contexts in which physiologically defined sleep is investigated, rather than assuming that sleep patterns reported in the populations studied to date represent cattle generally.

Methodological limitations also continue to restrict progress in the field. Although polysomnography remains the gold standard for sleep assessment, its complexity, cost, and labor requirements limit its application in large-scale studies.

Recent advances in accelerometry, wearable physiological monitoring, and automated classification offer promising opportunities for large-scale assessment; however, their validation depends on the reliability of the physiological reference against which they are compared. Although PSG remains the reference method for sleep-stage classification, noninvasive recordings in cattle are susceptible to signal artifacts, particularly those generated by rumination and masticatory muscle activity. Periods in which PSG signals cannot be reliably interpreted may therefore yield uncertain reference labels or require exclusion from validation datasets, limiting evaluation of alternative approaches across cattle’s full behavioral repertoire. Future methodological research should therefore prioritize robust signal acquisition and standardized classification criteria, and validation studies should (i) retain standing, lying wakefulness, drowsing and rumination as explicit classes rather than excluding them, (ii) report unclassifiable epochs as a separate category, (iii) report inter- and intra-observer reliability of the reference scoring for each state, (iv) report class-specific sensitivity, precision and agreement rather than overall accuracy alone, and (v) evaluate performance at the animal level and externally, in animals, farms or housing systems not used for model development. Only after addressing these methodological issues can researchers adequately establish the performance and generalizability of scalable monitoring systems.

To make future studies comparable and suitable for synthesis, we recommend that studies of sleep in cattle report, as a minimum: (i) breed, age, parity and physiological stage, criteria used to select animals (e.g., temperament screening), and housing and habituation procedures; (ii) electrode montage, sampling rate, filters, recording window and the amount of data lost; (iii) explicit operational criteria for each vigilance state, including quantitative thresholds for drowsing, and whether drowsing is included in total sleep; (iv) how rumination and other unscorable epochs were treated and how much time they represent; (v) the number of scorers, blinding to treatment and behavior, and inter- and intra-scorer agreement for each state; and (vi) for indirect methods, the reference standard, the prevalence of each class and the validation design. At present, the amount of data lost, the time spent ruminating or otherwise unscorable, scorer blinding and scorer agreement are each reported in no more than four of the twelve electrophysiological studies summarized in Supplementary Table S2.

Sample sizes have also been small, ranging from two or three animals in studies with implanted electrodes [6,24,26] to 19 cows [28], and only one study reported an a priori sample-size calculation, based on cytokine responses in humans rather than on sleep outcomes [44]. Between-animal variation is large: in nine lactating cows recorded for three days, daily REM and NREM sleep had standard deviations of 20 and 38 min, respectively [27]. With such variation, detecting a 10-min difference in daily REM sleep between two independent groups (α = 0.05; power = 0.80) would require approximately 63 cows per group. Within-animal designs, such as the crossover and repeated-measures designs already used in several studies [25,27,28], require far fewer animals and should be preferred, with sample sizes justified a priori from the expected within-animal variation.

Current evidence demonstrates that cattle exhibit distinct sleep stages and regulated sleep patterns. However, little is known about whether variation in sleep duration, stage distribution, fragmentation, or temporal organization influences productive performance, feed efficiency, metabolic regulation, immune competence, disease resilience, or welfare. The physiological links are plausible and supported by evidence from humans and laboratory animals, but direct evidence in cattle remains scarce. Importantly, the amount or pattern of sleep that constitutes adequate sleep for cattle has not been established, nor is there currently a validated threshold at which altered sleep indicates compromised welfare.

Future studies should therefore move beyond describing sleep architecture and test the biological consequences of experimentally or naturally occurring variation in sleep. This will require integrating physiologically validated sleep measurements with independent indicators of health, physiological function, behavior, welfare, and productive performance. Candidate outcomes include lying time and lying bouts, rumination time, feed intake and milk yield, inflammatory and metabolic markers [25,44], clinical health events and, where feasible, indicators of affective state, measured in the same animals and periods as sleep. Longitudinal and experimental designs will be particularly important for distinguishing associations from causal effects and for determining whether changes in sleep represent adaptive responses, indicators of biological challenge, or factors that themselves contribute to subsequent outcomes. Such evidence will be necessary to determine whether sleep can ultimately serve as a meaningful indicator or management-relevant biological variable in cattle production systems.

## 10. Conclusions

Sleep in cattle is a regulated, polyphasic state, but current knowledge rests on a small number of studies, mostly of dairy cows under controlled conditions. Physiological sleep, classified by PSG, must be distinguished from sleep-like behavior inferred from posture or movement, which in adult cattle cannot separate quiet wakefulness, drowsing and sleep. Because vigilance states cannot be classified during rumination with surface recordings, estimates of drowsing and NREM sleep depend on how rumination is handled. Sleep changes with lying deprivation and around calving, and sleep-related postures change with diet, bedding, outdoor access and relocation. Sleep changes with lying deprivation and around calving, and sleep-related postures change with diet, bedding, outdoor access and relocation, but no threshold of sleep duration or alteration indicating compromised welfare has been validated. Moreover, given the expected variation among animals, physiological stages, management systems, and environmental conditions, a single universal threshold separating adequate from inadequate sleep may not be biologically appropriate. These changes therefore cannot yet be interpreted in terms of welfare without considering their biological and production context. Progress will depend on establishing how reliably sleep can be measured and whether variation in sleep has measurable consequences for animal physiology, health, and welfare.

## Figures and Tables

**Table 1:** Non-rapid eye movement (NREM) sleep, rapid eye movement (REM) sleep, total sleep time (TST) and drowsing reported in electrophysiological studies of cattle (h:min per 24 h) ^1^.

Reference	Animals (*n*)	Recording; Rumination Handling ^2^	Condition	NREM	REM	TST	Drowsing
Ruckebusch [6]	Cows (3)	Implanted ECoG/EMG, 24 h; scored through rumination	Undisturbed	3:13	0:45	3:58	7:29
Ruckebusch [26]	Cows (3) ^3^	Implanted ECoG/EMG, continuous; scored through rumination	Control	3:48	0:23	4:11	–
			Recumbency prevented 14 h/d	2:54	0:26	3:20	–
			Recumbency prevented 20 h/d	4:05	0:06	4:11	–
			Recumbency prevented 22 h/d	4:36	0:00	4:36	–
			Recovery	5:48	1:09	6:58	–
Ruckebusch [24]	Cows (2)	Implanted ECoG/EMG, continuous; scored through rumination	12–4 d before calving	4:52	0:42	5:34	–
			2–10 d after calving	4:00	0:32	4:32	–
Hänninen et al. [12]	Calves, 3 months (6)	Surface PSG, 20 h; rumination scored separately (40% of observations)	Pair-housed; extrapolated ^4^	3:22	2:41	6:03	n.d.
Ternman et al. [27]	Lactating cows (9)	Surface PSG, 3 × 24 h; rumination scored separately (8.2 h/d)	Mean of 3 days	1:04	0:44	1:48	0:57
Ternman et al. [28]	Cows (19)	Surface PSG, 7 × 24 h; rumination scored separately (7.6–8.8 h/d)	Week −2 ^5^	1:26	0:50	2:17	1:05
			Week 2	0:54	0:34	1:28	0:38
			Week 7	1:12	0:47	1:59	0:45
			Week 13	1:27	0:43	2:09	0:41
			Week 22	1:30	0:44	2:14	0:49
			Week 37	1:25	0:52	2:17	0:55
			Week 45	1:19	0:43	2:02	0:42
Kull et al. [25]	Lactating cows (12)	Surface PSG, 24 h per condition; rumination not scored separately	Baseline	1:06	0:06	1:12	8:18
			Sleep deprivation ^6^	0:42	0:06	0:48	8:48
			Baseline	1:06	0:18	1:24	6:48
			Lying deprivation	0:18	0:06	0:24	5:06

^1^ Values are means reported in the original studies for 24-h recordings, either directly in h:min [6] or converted to h:min from percentages of 24 h [24,26], hours [25] or minutes [27,28]; only the values for calves [12] were extrapolated (footnote 4). Values are presented descriptively and are not intended for pooled estimation. TST = NREM + REM sleep; drowsing is a wakeful state and is not included in TST in any study. ^2^ The handling of epochs with rumination determines whether drowsing and NREM sleep occurring during rumination could be counted (Section 5). ^3^ Values from one of three cows shown in the original table (between-animal variation < 5%); recumbency was prevented for 14, 20 and 22 h per day over 8 weeks, with feeding restricted to the remaining hours. ^4^ Percentages of scored observations (NREM 14.0%, REM 11.2%) within approximately 15 h of readable data per calf, extrapolated to 24 h; calves were sedated for electrode attachment 24 h before recording. ^5^ Weeks relative to calving. ^6^ Gentle handling while lying was allowed; no vigilance state differed significantly from baseline. –, not reported; n.d., not distinguished from NREM sleep.

**Table 2:** Operational criteria used to classify vigilance states in electrophysiological studies of cattle.

State	EEG/ECoG	EMG (Neck)	EOG/Eyes	Behavior	Discriminating Features and Between-Study Differences
Alert wakefulness	Desynchronized low-voltage, high-frequency activity of mixed amplitude; movement artifacts common	High; varies with movement	Eyes open; blinks and eye movements	Standing or lying, head up and moving	Separated from REM sleep by muscle tone; tone may be relatively low during quiet wakefulness [29]
Drowsing	Mixture of low-voltage fast and high-voltage slow activity [6]; synchronized, with 10–30 Hz power 0.2–1.0 µV^2^/Hz [29]; frequent K-complexes [29]	Reduced relative to wakefulness; variable with movement	Upper eyelid relaxed; partial closure [6]	Mostly lying with head lifted and still [31]; low arousal threshold [6]	Quantitative threshold only in [29]; qualitative in [6,25]; not distinguished from NREM in calves [12]; not scored (N1/N2 instead) in [23]
NREM sleep	Synchronized high-amplitude, low-frequency (delta) activity; 10–30 Hz power < 0.2 µV^2^/Hz [29]; subdivided into N1–N3 in [23]	Reduced (persistent tone reported in [24])	Few eye movements (occasional in calves [12])	Lying, head lifted and still or held low	Arousal threshold approximately ten times higher than in drowsing [6]
REM sleep	Desynchronized, low-amplitude mixed-frequency activity resembling wakefulness	Atonia, occasional twitches	Bursts of rapid eye movements	Lying, head resting on flank or ground (not specific in adult cows [23,31])	Distinguished from wakefulness by atonia; rumination does not occur during REM sleep [28]
Rumination (surface electrodes)	Rhythmic high-amplitude 2–5 Hz chewing artifact obscuring all channels [12,29]	Rhythmic artifact	Artifact	Rhythmic chewing not related to eating	Underlying state not classifiable: scored as a separate class [12,27,28], removed [13,23] or not separated [25]; implanted electrodes allowed drowsing or NREM sleep to be scored during rumination [6]

ECoG: electrocorticography; EEG: electroencephalography; EMG: electromyography; EOG: electrooculography. N1–N3: stages of NREM sleep as defined for human sleep scoring.

**Table 3:** Characteristics, methodological approaches, and experimental contexts of the 22 primary studies assessing physiological sleep or sleep-like behavior in cattle.

Reference	Animals (*n*)	Housing	Study Focus	Recordings per Animal	PSG	Rumination
Implanted electrodes						
Merrick [5]	Steers (2)	Confined	Resting states	24 h	Yes	NR
Ruckebusch [6]	Adult cows (3)	Stalls	Vigilance states	2–3 × 24 h/week	Yes	Scored
Ruckebusch [26]	Dry cows, 4 years (3)	Stall	Lying prevention	~10 weeks, continuous	Yes	Scored
Ruckebusch [24]	Cows around calving (2)	Stall	Parturition	~6 weeks, continuous	Yes	Timed (jaw sensor)
Surface electrodes (noninvasive PSG)						
Hänninen [12]	Calves, 3 months (6)	Pair pens	Behavior vs. PSG	1 × 20 h	Reference	Separate class (40%)
Ternman [29]	Dry cows (8)	Single pens	PSG feasibility	1 × ~5.7 h	Yes	Not analyzable
Ternman [31]	Lactating and dry cows (13)	Single pens	Behavior vs. PSG	1 × ~4.4 h	Reference	Identified behaviorally
Ternman [27]	Lactating cows (9)	Single pens	First-night effect	3 × 24 h	Yes	Separate class (8.2 h/d)
Ternman [28]	Cows, week −2 to 45 of lactation (19)	Single pens	Lactation cycle	7 × 24 h	Yes	Separate class (7.6–8.8 h/d)
Kull [25]	Lactating cows (12)	Box stalls	Lying/sleep deprivation	4 × 24 h	Yes	Not separated
Proudfoot [44]	As [25] (12)	Box stalls	Metabolism, inflammation	As [25]	Data of [25]	Not separated
Hunter [23]	Dry cows (10)	Indoor/pasture pens	Posture vs. PSG	Repeated 10 h	Reference	Removed
Hunter [45]	As [23] (10)	Indoor/pasture pens	HR/HRV by sleep stage	Repeated ≤10 h	Yes	Removed
Hunter [13]	As [23] (12)	Indoor/pasture pens	ML sleep staging	Repeated 10 h	Reference	Removed
Posture recorded by video						
Dan [37]	Beef fattening cattle, ~20 months (8)	Group pens	Bedding cleanliness	8 × 24 h	No	Not addressed
Huricha [38]	Dry cows (5)	Tie-stall, outdoor access	Outdoor access	10 × 24 h	No	Not addressed
Posture recorded by accelerometers						
Hokkanen [35]	Calves, ~1 month (10)	Group pen	Posture prediction	1 × 24 h	No (video)	Not separated
Klefot [42]	Lactating cows (4)	Tie-stall	Sensor validation	2 × 24 h	No (observation)	Counted as wake/drowsy
Fukasawa [43]	Pregnant beef cows (7)	Tie-stall	Sensor validation	9–10 × 12 h	No (video)	Not addressed
Fukasawa [41]	Pregnant beef cows (7)	Tie-stall	Relocation	17 × 24 h	No	Not addressed
Fukasawa [33]	Pregnant beef cows (6)	Tie-stall	Diet	6 × 23.5 h	No	Measured (jaw sensor)
Fukasawa [36]	Heifers, 0.5–23 months (15)	Single, then group pens	Age	8 × 24 h	No	Not addressed

Reference: first author and reference number; n = number of animals analyzed (dairy breeds unless stated; breed not reported in [6,24,26]). The same animals were used in [25,44] and in [13,23,45]; five recordings of [29] were re-scored in [31]. PSG: Yes, states classified electrophysiologically; Reference, PSG used to validate behavior, posture or a heart rate and EMG model; No, no physiological measurement (validation reference in parentheses). Rumination: Scored, states classified during rumination with implanted electrodes; Removed, rumination and standing epochs removed before analysis. HR: heart rate; HRV: heart rate variability; ML: machine learning; NR: not reported. Details are given in Supplementary Table S1.

**Table 4:** Validation of behavioral, accelerometer and physiological approaches for estimating sleep in cattle.

Reference	Index Method	Reference Standard	Animals	States Excluded Before Validation	Validation Level	Main Results
Hänninen et al. [12]	Behavior (posture, eyes)	PSG	5 calves, 3 months	Rumination (40% of observations)	Within animals; no cross-validation	Sleep: Se 78%, Sp 82%; NREM: Se 54%, Sp 89%; REM: Se 49%, Sp 90%; κ 0.31–0.71
Ternman et al. [31]	Behavior (posture)	PSG	13 cows	Not reported	Within animals	Behavioral NREM: Se 81%, Sp 6%; REM: Se 70%, Sp 41%; TST overestimated (median 141 vs. 37 min per session)
Hunter et al. [23]	Lying posture (video)	PSG	10 dry cows (indoor, pasture)	Rumination, standing	Pooled epochs, by site	Tucked posture → REM: PPV 45% (indoor), 69% (pasture); head low → N3: PPV 4–6%
Hokkanen et al. [35]	Neck accelerometer; SVM	Video postures	10 calves, ~1 month	None reported	Leave-one-calf-out	Total sleep time 90%; 45% of lying-awake epochs classified as NREM; NREM over- and REM underestimated
Klefot et al. [42]	Neck accelerometer; LDA	Direct behavioral observation	4 lactating cows	Uncertain segments, transitions, drowsy behavior	Segment-level bootstrap	Wake 93.7%, sleep-like behavior 92.2% correctly classified
Fukasawa et al. [43]	Occipital accelerometer	Video sleeping posture	7 cows	–	Per night	κ > 0.80 in 91.5% of nights (posture detection only)
Hunter et al. [13]	HR, HRV, neck EMG; neural network, random forest	PSG	12 dry cows	Rumination, standing	10-fold cross-validation of randomly split epochs	Overall accuracy 82%; 72% of epochs awake; class-specific recall not reported

Se: sensitivity; Sp: specificity; PPV: positive predictive value; κ: Cohen’s kappa; TST: total sleep time; SVM: support vector machine; LDA: linear discriminant analysis; HR: heart rate; HRV: heart rate variability. Details are given in Supplementary Table S3.

**Table 5:** Comparison of approaches used to assess physiological sleep or sleep-like behavior in cattle.

Approach	Information Recorded	Sleep Inference	Main Strengths	Main Limitations
Behavioral observation	Posture, movement, resting behavior	Sleep-like behavior	Low cost; applicable under field conditions	Cannot reliably distinguish quiet wakefulness, drowsing, NREM, and REM in adult cattle
Accelerometry	Movement and body/head position	Sleep-like behavior based on movement and posture	Automated; continuous; scalable	Shares limitations of behavioral observation; immobility does not necessarily indicate physiological sleep
HR/HRV monitoring *	Heart rate and autonomic variation	Indirect inference of vigilance state	Continuous physiological monitoring; compatible with wearable systems	Affected by non-sleep physiological processes; evidence for sleep classification in cattle remains limited and has involved pre-processed data
Polysomnography	EEG, EOG, and EMG	Physiological classification of vigilance states	Reference method for distinguishing wakefulness, drowsiness, NREM, and REM	Technically demanding; limited scalability; susceptible to signal artifacts, particularly during rumination
Machine learning (analytical approach)	Any of the signals above, alone or combined	Depends on the input signals and reference labels	Automated integration of several signals; scalable	Not an independent measurement method; performance bounded by the reference labels and the validation design; in cattle validated only at the epoch level, with standing and rumination excluded [13]

HR: heart rate; HRV: heart rate variability; EEG: electroencephalography; EMG: electromyography; EOG: electrooculography. * Sleep-stage classification from HR and HRV has so far relied on machine-learning models [13].

**Table 6:** Factors tested for effects on sleep or sleep-related behavior in cattle.

Factor	Reference (*n*)	Sleep Measure	Design	Effect (Direction and Magnitude)	Other Outcomes
Prevention of recumbency	[26] (3)	Implanted PSG	Within animals, 8 weeks	REM almost abolished at 22 h/d; NREM 12–19% vs. 16% of 24 h; rebound to ~2× for 4 d	Fatigue, weight loss (feeding time also restricted)
	[25] (12)	Surface PSG	Crossover, 24 h	NREM 1.1 → 0.3 h (*p* = 0.02); REM 0.3 → 0.1 h (*p* = 0.08); wakefulness 15.9 → 18.5 h	Milk yield ↓ on days 1–2 [25]; TNF, IL1B, IL6 expression ↑ [44]
Gentle handling (sleep deprivation)	[25] (12)	Surface PSG	Crossover, 24 h	No change in any vigilance state	No change in cytokines [44] or milk yield [25]
Calving and early lactation	[24] (2)	Implanted PSG	Within animals, observational	Sleep ↓ ~50% in the 24 h around calving; NREM 20.3 → 16.7%, REM 2.9 → 2.2% of 24 h after calving	Feed intake and rumination ↑
	[28] (19)	Surface PSG	Longitudinal, 7 × 24 h	Week 2 vs. week −2: REM 50 → 34 min (*p* = 0.01); NREM 86 → 54 min (*p* = 0.05–0.06); drowsing 65 → 38 min	–
Recording day	[27] (9)	Surface PSG	3 consecutive days	No effect on daily totals	–
Time of day	[27,28]	Surface PSG	Observational	NREM, REM and rumination predominantly at night	–
Suckling and calf separation	[24] (2)	Implanted PSG	Observational	Sleep ↓ (up to 50% for 48 h)	Feeding ↓
Relocation to a new barn	[41] (7)	Sleep posture (accelerometer)	Longitudinal, 35 d	Lowest on day 1 (55 min/d); stable (~65 min/d) after about one week	–
Concentrate-rich diet	[33] (6)	Sleep posture (accelerometer)	Crossover	96 vs. 69 min/d (*p* < 0.01)	Rumination 143 vs. 405 min/d
Bedding cleaned	[37] (8)	Sleep-related postures (video)	Two periods, treatments swapped	Total unchanged; tucked ↓, extended ↑	Cortisol and serotonin unchanged
Outdoor access, 1 h/d	[38] (5)	REM sleeping posture (video)	Sequential, not randomized	1.51 → 1.40 h/d (*p* = 0.047), proportional to lying time	T-cell proliferation ↑
Age (0.5–23 months)	[36] (15)	Sleep-like posture (accelerometer)	Longitudinal	Decreases with age; stable (~60 min/d) after 12 months	–
Housing and site (indoor vs. pasture)	[23] (10)	Surface PSG	Cross-sectional; confounded by breed, pregnancy and season	Asleep 43% vs. 18% of non-ruminating lying time	–

↓ decrease; ↑ increase. Posture-based measures indicate sleep-related behavior, not physiologically defined sleep.

## Data Availability

No new data were created or analyzed in this study. Data sharing is not applicable to this article.

## Supplementary Materials

The following supporting information can be downloaded at: https://www.mdpi.com/article/10.3390/ani16203279/s1, Table S1: Characteristics of the primary studies on sleep and sleep-related behavior in cattle; Table S2: Acquisition, scoring and reporting in electrophysiological studies of sleep in cattle. (a) Acquisition and scoring, and (b) Reporting completeness.; Table S3: Validation of behavioral, accelerometer and physiological approaches for estimating sleep in cattle.
